# Molecular Evolution of the Influenza A Virus Non-structural Protein 1 in Interspecies Transmission and Adaptation

**DOI:** 10.3389/fmicb.2021.693204

**Published:** 2021-10-04

**Authors:** Danyel Evseev, Katharine E. Magor

**Affiliations:** Department of Biological Sciences, Li Ka Shing Institute of Virology, University of Alberta, Edmonton, AB, Canada

**Keywords:** evolution, adaptation, interferon, CPSF, host, species, RIG-I, innate

## Abstract

The non-structural protein 1 (NS1) of influenza A viruses plays important roles in viral fitness and in the process of interspecies adaptation. It is one of the most polymorphic and mutation-tolerant proteins of the influenza A genome, but its evolutionary patterns in different host species and the selective pressures that underlie them are hard to define. In this review, we highlight some of the species-specific molecular signatures apparent in different NS1 proteins and discuss two functions of NS1 in the process of viral adaptation to new host species. First, we consider the ability of NS1 proteins to broadly suppress host protein expression through interaction with CPSF4. This NS1 function can be spontaneously lost and regained through mutation and must be balanced against the need for host co-factors to aid efficient viral replication. Evidence suggests that this function of NS1 may be selectively lost in the initial stages of viral adaptation to some new host species. Second, we explore the ability of NS1 proteins to inhibit antiviral interferon signaling, an essential function for viral replication without which the virus is severely attenuated in any host. Innate immune suppression by NS1 not only enables viral replication in tissues, but also dampens the adaptive immune response and immunological memory. NS1 proteins suppress interferon signaling and effector functions through a variety of protein-protein interactions that may differ from host to host but must achieve similar goals. The multifunctional influenza A virus NS1 protein is highly plastic, highly versatile, and demonstrates a diversity of context-dependent solutions to the problem of interspecies adaptation.

## Introduction

The human-animal interface is impacted by ecological change and human encroachment, and zoonotic spillover continues to challenge human health and agriculture. The adaptation of viruses to new host species remains a relevant subject of investigation, and influenza A viruses (IAVs) offer many interesting examples. Recent strides have been made in understanding the evolutionary pressures that drive IAV host adaptation and the pathways to development of airborne transmission in new hosts. For example, the evolution of the viral surface glycoproteins and their roles in cross-species transmission are well known and adaptation of the viral polymerase components to new cellular environments is necessary for efficient viral replication (Rogers and Paulson, 1983; De Graaf and Fouchier, 2014; Long et al., 2019). In comparison, the selective pressures that drive evolution of the principle IAV immune antagonist, the non-structural protein 1 (NS1), have been more elusive. NS1 is an essential virulence factor with myriad immune-antagonistic functions and diverse sequence variants. In this review we aim to highlight what is known about the role of NS1 in interspecies adaptation and the changes that accompany this process. A general summary of host-specific signatures in the NS1 protein is followed by a detailed discussion of two specific functions – the suppression of host protein synthesis and the inhibition of antiviral interferon signaling by the host retinoic acid-inducible gene I (RIG-I) pathway.

## Waterfowl are the Natural Hosts of Influenza a Virus

Influenza A viruses have a common evolutionary ancestry in aquatic birds (Webster et al., 1992; Yoon et al., 2014). Phylogenetic analyses of the individual gene segments suggest that all circulating human IAVs have ancestors in the avian lineage (Okazaki et al., 1989; Gammelin et al., 1990; Gorman et al., 1991). The same is true of IAVs in other mammals (Guo et al., 1992; Li et al., 2010; Ciminski et al., 2020). Most of the known influenza A HA and NA subtypes circulate in wild ducks (Yoon et al., 2014), with the exception of H13 and H16 subtypes, which circulate primarily in gulls (*Laridae* family; Kawaoka et al., 1988; Chambers et al., 1989). Several new subtypes of influenza A virus circulating in bats have recently been discovered (Tong et al., 2013), which appear to have been evolving in isolation for a long time but presumably also with avian ancestors (Mehle, 2014; Ciminski et al., 2020).

As the reservoir host, ducks have co-evolved with avian IAVs to a state in which a high degree of viral replication is tolerated and damage to the host is limited (Webster et al., 1992). The diverse strains that circulate in the duck reservoir are mainly low-pathogenicity and replicate in their intestinal tracts (Kim et al., 2009). Mallard ducks (*Anas platyrhynchos*) have a higher prevalence of IAV infection than other wild birds, harbour the low-pathogenicity strains without signs of disease, and shed them into water (Kida et al., 1980; Runstadler et al., 2007; Jourdain et al., 2010) where they appear to be stable for months (Stallknecht et al., 1990a,b). In their seasonal migrations, ducks circulate influenza viruses along their migration pathways (Hulse-Post et al., 2005; Kilpatrick et al., 2006; Marchenko et al., 2012). The global patterns of influenza A virus prevalence in wild birds are reviewed by Olsen et al. (2006).

Where wild migratory birds come into contact with domesticated fowl, other animals, or humans, zoonotic spillover can occur. Periodically, and many times over the course of history, IAVs have crossed over from wild birds into other species and occasionally have established stably transmissible lineages. All recorded human influenza pandemics have begun with the introduction of new avian genes into mammalian viruses by reassortment (Smith et al., 2009; Guan et al., 2010).

## Molecular Determinants of Host Adaptation of Influenza a Viruses

Influenza A virus genes accumulate distinct polymorphisms over time in different species. For example, the PB2, PA, NP, M, and NS genes have diverged into distinct lineages in avian vs. human hosts (Bean, 1984; Okazaki et al., 1989; Gammelin et al., 1990; Gorman et al., 1990a,b, 1991; Ito et al., 1991; Furuse et al., 2009). Analyses comparing rates of synonymous vs. non-synonymous substitutions in several IAV genes demonstrated that viruses in terrestrial poultry, pigs, and humans accumulate amino acid mutations faster than those in wild aquatic birds, suggesting that they are adapting more rapidly to these relatively new hosts (Gorman et al., 1990b, 1991; Sugita et al., 1991; Shu et al., 1993; Furuse et al., 2009). While overall nucleotide substitution rates in IAVs are comparably high across avian and mammalian hosts, it appears that stronger selective pressures are acting on multiple viral genes in mammals, reflecting the process of adaptation from avian ancestors (Gorman et al., 1990a; Chen and Holmes, 2006; Furuse et al., 2009). However, this does not necessarily mean that avian influenza viruses in their wild reservoir have achieved an evolutionary stasis. Rather, they continue to evolve over time undergoing periodic evolutionary bottlenecks and continual selective turnover (Chen and Holmes, 2006).

With the accumulation of viral sequences and functional studies, some important molecular determinants of host adaptation have become apparent, for example, the alteration of HA binding specificity and NA activity to accommodate the sialic acid linkages that predominate in mammalian vs. avian airways (Rogers and Paulson, 1983; Matrosovich et al., 1999; De Graaf and Fouchier, 2014; Long et al., 2019; de Vries et al., 2020). The ribonucleoprotein components NP, PA, and PB2 acquire a plurality of species-specific amino acid changes in mammalian hosts (Gorman et al., 1991; Chen and Holmes, 2006; Finkelstein et al., 2007). Since the host innate immune system is an important hurdle for efficient viral establishment and transmission, it follows that adaptations in the main immune antagonist of the virus, the NS1 protein, must also be important in viral evolution and interspecies transmission.

## The IAV Ns1 Protein

Influenza A virus NS1 is a virulence factor that is expressed in host cells but not packaged into the virion. It facilitates viral RNA synthesis and replication by recruiting host factors while simultaneously shutting down host cell innate immune signaling and antiviral effector functions in multiple ways, through multiple protein-protein interactions.

**Figure 1:**
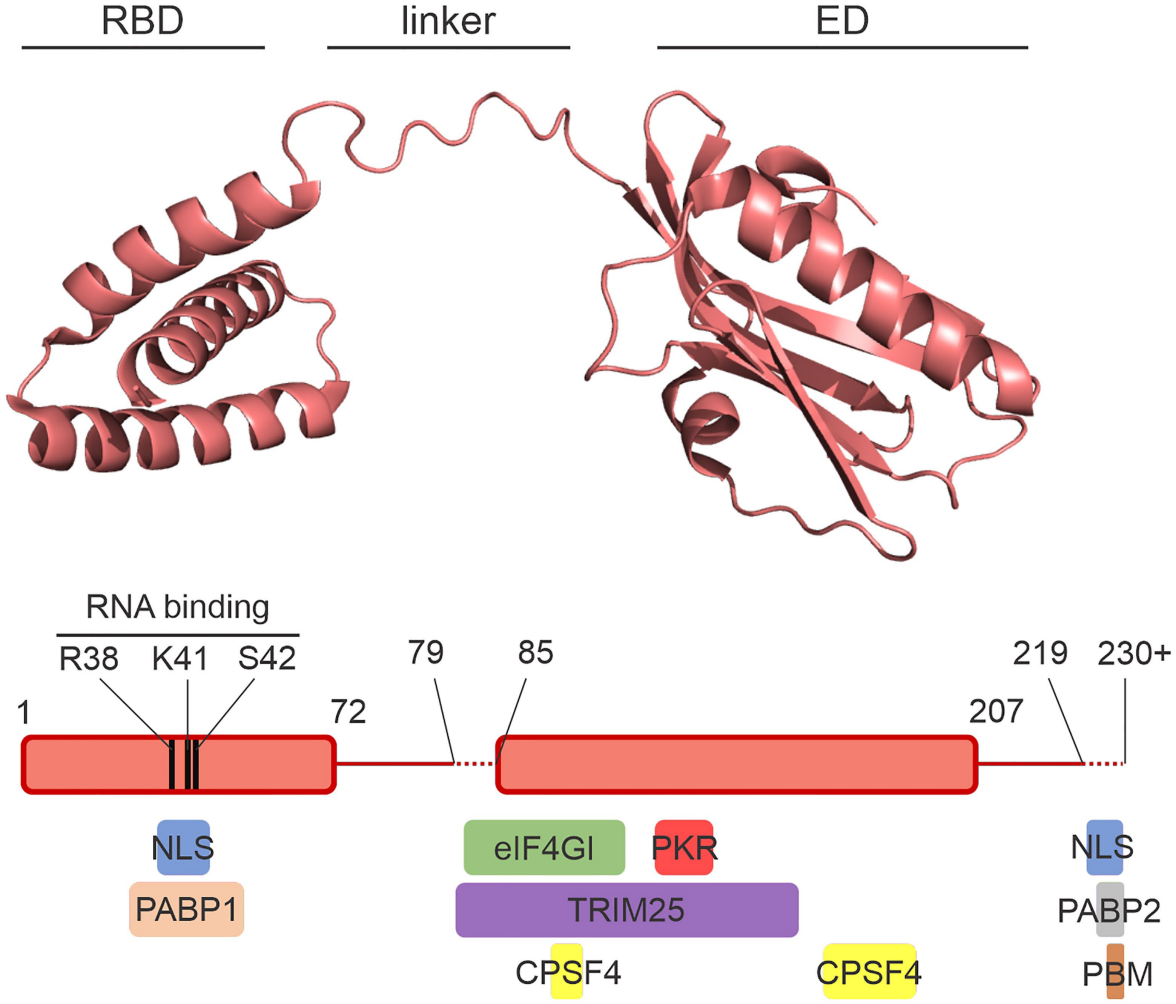
The NS1 protein. A crystal structure of the NS1 protein from A/Vietnam/1196/2004 (H5N1) is shown with the RNA-binding domain (RBD), the effector domain (ED), and the flexible interdomain linker labeled. The diagram below shows the amino acid limits of the domains. The variable lengths of the linker and the structurally disordered C-terminal tail are represented with dashed lines. Colored boxes below the schematic illustrate the approximate regions of NS1 involved in interactions with a select set of host proteins. NLS, nuclear localization sequence; PBM, PDZ-binding motif. Image was prepared with PyMol using the crystal structure from Mitra et al. (2019; PDB ID: 6O01).

This NS1 is a small protein of approximately 230 amino acids composed of two domains joined by a flexible linker region (Bornholdt and Prasad, 2008; Figure 1). The smaller N-terminal domain consists of three alpha-helices between residues 1 and 72 and is called the RNA-binding domain (RBD). The C-terminal effector domain (ED) comprises residues 85–207. The very C-terminal tail is variable in length from strain to strain and is structurally disordered (Soubies et al., 2013). Because the NS1 protein is so small and yet participates in many protein-protein interactions, many interface regions overlap on the NS1 structure (Min et al., 2007).

NS1 protein facilitates viral replication in several ways. NS1 temporally regulates viral RNA synthesis in host cells (Min et al., 2007). It promotes the translation of viral mRNA by recruiting host eukaryotic initiation factor 4GI (eIF4GI; Enami et al., 1994; de la Luna et al., 1995; Aragón et al., 2000; Zhou et al., 2010) and poly(A)-binding protein I (PABP1) to viral translation initiation complexes (Burgui et al., 2003). Simultaneously, nuclear fractions of NS1 suppress host pre-mRNA maturation by binding to and inhibiting cleavage and polyadenylation specificity factor 4 (CSPF4, 30kDa subunit) and poly(A)-binding protein II (PABP2; Nemeroff et al., 1998; Li et al., 2001; Noah et al., 2003; Das et al., 2008; Kuo and Krug, 2009; Hale et al., 2010; Khaperskyy and McCormick, 2015). Its most important role, however, seems to be the inhibition of type I interferon (IFN) signaling and effector functions (Hale et al., 2008b). This is because recombinant IAVs lacking NS1 or containing large NS1 deletions replicate normally in IFN-deficient cells and animals, but not in IFN-competent organisms – they are severely attenuated in the presence of interferon (Egorov et al., 1998; García-Sastre et al., 1998; Kochs et al., 2007; Zhu et al., 2008; Soubies et al., 2010b; Wang et al., 2019). Del-NS1 viruses can adapt for replication in interferon-deficient cells *via* combinations of point mutations in other genes, but they are non-pathogenic in mice while also being highly immunogenic (Wang et al., 2019). Therefore, NS1 is necessary for IAVs to evade the innate immune system and replicate effectively in all hosts. Even among full-length NS1 proteins, sequence differences that affect the degree of interferon inhibition can influence viral replication and disease severity (Meunier and von Messling, 2011).

In human cells, NS1 proteins oppose interferon induction by inhibiting the RIG-I viral sensing pathway (Gack et al., 2009; Rajsbaum et al., 2012; Koliopoulos et al., 2018). They can also directly inhibit the actions of antiviral interferon-stimulated genes. NS1 can interact directly with and suppress host antiviral effector protein kinase R (PKR), which blocks viral mRNA translation by phosphorylating eukaryotic translation initiation factor eIF2α (Li et al., 2006; Min et al., 2007). NS1 prevents the activation of another interferon-stimulated gene, RNase L, by binding and sequestering any free double stranded RNA (dsRNA) generated during viral replication. RNase L is a ribonuclease that destroys all RNA in the cell when activated by 2'-5'-oligoadenylate synthetase (OAS). OAS detects the presence of (viral) double-stranded RNA, but with a weak affinity that can be outcompeted by NS1 (Min and Krug, 2006; Krug, 2014). For excellent comprehensive reviews of the many functions of NS1 in viral replication, see Hale et al. (2008b) and Ayllon and García-Sastre (2015).

## NS1 Protein Phylogeny and Host Adaptation Overview

Apart from the surface antigens HA and NA, NS1 is the most polymorphic protein of IAVs (Chen and Holmes, 2006; Kumar et al., 2006; Obenauer et al., 2006). A genome-wide mutagenesis study revealed that the HA and NS1 genes are particularly tolerant to mutation, and that most of the mutation-tolerant sites in NS1 are surface-exposed (Heaton et al., 2013). IAV NS1 proteins fall into two phylogenetic clades that diverged early in IAV evolution, termed allele A and allele B (Treanor et al., 1989; Webster et al., 1992; Xu et al., 2014). Between members of a single allele, whether A or B, there is approximately 90% amino acid sequence conservation; between alleles, NS1 proteins share approximately 72% identity (Treanor et al., 1989). With rare exceptions, all known mammalian strain NS1 proteins belong to allele A, while avian strains can possess either allele A or allele B NS1 proteins (Ludwig et al., 1991; Guo et al., 1992; Zohari et al., 2008; Xu et al., 2014). Within each allele there are species-specific and geographically isolated lineages (Xu et al., 2014), but allele A and B strains can also be found co-circulating in wild birds in the same geographic regions at the same time (Zohari et al., 2008). Dugan et al. (2008) proposed that the maintenance of both alleles in the natural reservoir suggest that they are under some form of balancing selection.

The observation that B-allele NS segments are virtually restricted to birds initially led to the hypothesis that the NS1 proteins belonging to that allele are less well-adapted to replication in mammalian hosts. This was supported by the fact that a recombinant influenza A/Udorn/1972 (H3N2) bearing a B-allele NS1 gene was attenuated in the respiratory tracts of squirrel monkeys compared to the wild-type virus (Treanor et al., 1989). Similarly, an allele A NS1 from A/Mink/Sweden/1984 (H10N4) was more potent at inhibiting an interferon-stimulated response element reporter system than B-allele NS1 from A/Chicken/Germany/N/1949 (H10N7) in human A549 cells (Zohari et al., 2010). However, Turnbull et al. (2016) found that swapping NS alleles in the context of human H1N1 and H3N2 viruses did not attenuate them in various mammalian cell lines or in mice, and did not reduce their ability to inhibit interferon signaling. Furthermore, replacing the endogenous allele A NS segment in A/FPV/Rostock/1934 (H7N1) with the NS segment from A/Goose/Guangdong/1/1996 (H5N1; allele B NS1) improved replication efficiency in human and murine cells, and increased pathogenicity in live mice (Ma et al., 2010). Thus, the reasons for the phylogenetic segregation of these two NS1 lineages are currently unclear.

Phylogenetically, the NS segments of all currently circulating human and swine viruses derive from a single lineage that traces back to the 1918 pandemic H1N1 strain. Since then, human and swine NS1 proteins have been evolving separately from the avian lineages and have accumulated species-specific substitutions in both protein domains and in the inter-domain linker region (systematically analyzed by Vasin et al., 2017). The species-associated polymorphisms with functional consequences that have been tested experimentally are summarized in Table 1. The dimerization interface of the N-terminal RBD of NS1 depends on six amino acids (Liu et al., 1997; Wang et al., 1999) that are universally conserved in all IAV strains. RNA binding depends, in part, on residues R35, R38, and K41, which also form a nuclear localization sequence essential for interaction of NS1 with importin α (Greenspan et al., 1988; Melén et al., 2007). R35 and R38 are universally conserved, as is K41, with the exception of the human H3N2 lineage, in which a K41R mutation was observed in sequenced isolates from 1974 onward (Vasin et al., 2017). In general, the dates given for the emergence of lineage-specific polymorphisms reflect their appearance in the sequences of dated isolates and not necessarily the exact years of their appearance. An R44K substitution was detected in the seasonal human H1N1 NS1 lineage in 1935 and independently in the classical swine lineage in 1971. However, in the seasonal H2N2 viruses that arose out of the seasonal H1N1 lineage in 1957, and the H3N2 lineage that replaced them in 1968, this amino acid has reverted to the original R44. The functional consequences of this polymorphism are unknown, but in combination with mutations V18A and S195P it yields an attenuated, temperature-sensitive virus (Garaigorta et al., 2005). Several other substitutions exist in the NS1 RBD that only occur in the human lineage – R21Q, F22V, A23V, and A60V – which fall outside of known interaction interfaces and whose functional consequences are unknown. A serine at position 42 (S42) has been linked to pathogenicity in mice (Jiao et al., 2008). This amino acid participates directly in RNA binding by the RBD (Cheng et al., 2009) and is consequently conserved in the majority of human, swine, and avian strains in the NCBI (National Center for Biotechnology Information) influenza database.

**Table 1:** Species-associated NS1 polymorphisms with investigated functional roles.

Residue positions	Amino acid polymorphisms
55	64% of avian strains encode E55, 31% encode R55, and 3% encode K55. Among human and swine strains, 55% encode E55, 43% encode K55, and 1.3% encode R55. Residue E55 enhances interferon inhibition in mammalian models (Murakami et al., 2008; Li et al., 2018).
80–84	Deletion in linker of avian H5N1 NS1 proteins is associated with enhanced virulence in chickens and mice (Long et al., 2008; Trapp et al., 2014).
92	Glutamic acid (E92) in avian H5N1 strains enhances pathogenicity in experimentally infected mammals. Most other known strains encode an aspartic acid (D92; Seo et al., 2002; Lipatov et al., 2005).
215	38% of human and swine strains encode T215 compared to 2% of avian strains. T215 is phosphorylated by host CDK/ERK kinases with unknown functional consequences. Replacement of T215 in a human virus with the avian-like P215 did not reduce replication efficiency (Hale et al., 2009; Hsiang et al., 2012).
226–230	The consensus sequence for avian strains is ESxV and for human strains it is RSxV. NS1 proteins with ESxV interact with host PDZ proteins that promote cell survival and disrupt tight junctions in mammalian cells (Liu et al., 2010; Golebiewski et al., 2011).

A polymorphic residue at position 55 in the NS1 RBD may be associated with host adaptation. In all the avian influenza A NS1 sequences in the NCBI influenza database (22,285 full-length sequences, 7,181 nonredundant on May 23, 2021), 64% encode a glutamic acid at this position (E55), 31% encode an arginine (R55), and 3% encode a lysine (K55). By contrast, in human and swine influenza A NS1 sequences (46,737 full-length sequences, 9,508 nonredundant on May 23, 2021), 55% encode E55, 43% encode K55, and 1.3% encode R55. Li et al. (2018) found that having a glutamic acid (E) instead of a lysine (K) at position 55 in the NS1 of H5N1 viruses enhanced viral replication and interferon suppression in human A549 cells. The A/Puerto Rico/8/1934 (H1N1) strain (PR8), which has been propagated in mice in laboratories around the world since the 1940s, exists as several variants, some of which encode K55 in the NS1 protein, and some of which encode E55. Several groups have investigated the contribution of this polymorphism to viral replication and virulence. Liedmann et al. (2014) compared two PR8 variants with different passage histories that had different pathogenicity in mice. The two variants differed by multiple substitutions across eight viral proteins, including K55E in NS1. The PR8 variant encoding NS1-K55 was lethal to mice and induced less interferon, but the authors found that swapping the NS gene did not alter the viral phenotype. Instead, the changes in pathogenicity and immune induction were attributed to changes in the PB1 and PA proteins. On the other hand, in their investigation of several PR8-based recombinant vaccine viruses, Murakami et al. (2008) found that a glutamic acid at position 55 in PR8 NS1 enhanced interferon inhibition and growth kinetics. It is possible that a lysine at this position, which predominates in human samples, is a site of post-translational modification, but more research is needed to establish if that is the case, and to investigate the consequences of these substitutions in avian hosts.

A flexible 12-aa linker (residues 73–84) joins the N-terminal RBD to the C-terminal effector domain. Its flexibility allows the domains to move independently and to adopt several different conformations relative to each other (Carrillo et al., 2014). A five-amino-acid truncation in this region is a known virulence determinant that arose in avian H5N1 viruses that reassorted with the progeny of A/Goose/Guangdong/1/1996 (H5N1) and became prevalent in highly pathogenic avian influenza (HPAI) H5N1 strains circulating in the first decade of the 2000s (Guan et al., 2004; Li et al., 2004; Zhou et al., 2006). Although shortening of the flexible linker does not appear to restrict the conformations that the two globular domains of NS1 can adopt *in vitro* (Mitra et al., 2019), the presence of this truncation increases H5N1 strain virulence in chickens and mice (Long et al., 2008). An avian H9N2 virus isolated from backyard poultry in Pakistan in 2013 was found to encode an H5N1-like NS1 bearing this deletion, acquired through reassortment (Munir et al., 2013). Engineering this truncation into the NS1 protein of an avian H1N1 virus also enhanced pathogenicity in experimentally infected chickens, with the recombinant mutant virus inducing more interferon and proinflammatory cytokine transcription in lungs compared to wild-type (Trapp et al., 2014). This truncation overlaps the region of NS1 that is responsible for recruiting host translation factor eIF4GI to promote viral mRNA translation (residues 81–113; Enami et al., 1994; de la Luna et al., 1995; Aragón et al., 2000; Zhou et al., 2010). Zhou et al. (2010) found that re-inserting amino acids into a naturally truncated H5N1 NS1 in a recombinant virus background attenuated both pathogenicity and replication in in live chickens and embryonated chicken eggs. In strains bearing a full-length inter-domain linker, there exists a species-associated polymorphism within the eIF4GI-binding motif: avian NS1 proteins encode isoleucine-81 (I81) and human NS1 proteins encode methionine-81 (M81; Finkelstein et al., 2007).

Several other lineage-specific substitutions have occurred in the eIF4GI-binding motif. In the classical swine lineage, a T91A substitution was replaced by A91S in the 2009 swine-origin pandemic strains (H1N1)pdm09. In human seasonal strains, whose NS1 proteins originate from the 1918 pandemic strain, a leucine (L) at position 95 was replaced with isoleucine (I), which became I95T in seasonal H3N2 strains since 1975, and I95V in seasonal human H1N1 strains since 2001 (Vasin et al., 2017).

Avian H5N1 strains between 1957 and 2001 acquired a glutamic acid at position 92 (E92) that enhanced pathogenicity in experimentally infected pigs (Seo et al., 2002). In fact, the E92-encoding NS1 from A/Hong Kong/156/1997 (H5N1) not only enhanced virulence of a recombinant virus in pigs, but also conferred greater interferon resistance, allowing the recombinant virus to replicate in cells pre-treated with IFN-α, IFN-γ or TNF-α. However, several NS1 proteins from descendant H5N1/2001 avian isolates also conferred this interferon resistance while encoding D92 and a five amino acid truncation in the interdomain linker. Rather than resistance to interferon pre-treatment, residue E92 appears to confer pathogenicity, as Lipatov et al. (2005) demonstrated in mice and Yucatan miniature pigs. Recombinant PR8 viruses encoding the H5N1/1997 NS segment induced severe disease and inflammation while those encoding an E92D mutation or an H5N1/2001 NS segment did not. In mouse lungs, the H5N1/97 NS-containing virus induced more pro-inflammatory cytokines and suppressed the anti-inflammatory cytokine IL10, compared to viruses with H5N1 NS1 proteins encoding D92. All other known human, swine, avian and equine IAVs have an aspartic acid at this position (D92; Li et al., 2004; Jiao et al., 2008).

Several conserved residues of NS1 are sites of covalent modification. A proline-to-tyrosine substitution (P215T) in the human NS1 lineage has been linked to phosphorylation (Finkelstein et al., 2007; Hale et al., 2009). In avian NS1 proteins, proline-215 lies in a SRC homology 3 (SH3) binding motif, PLPP (Finkelstein et al., 2007; Shin et al., 2007). In many human NS1 proteins this motif became PLTP (which still conforms to the PxxP consensus) and threonine-215 is phosphorylated by cyclin-dependent kinases (CDKs) and extracellular signal-regulated kinases (ERKs) in human cells (Hale et al., 2009; Hsiang et al., 2012). Introducing a T215A mutation attenuated the human A/Udorn/1972 (H3N2), but replacement of T215 with the avian-like P215 did not reduce replication efficiency in human Calu-3 cells and MDCK cells (Hsiang et al., 2012). Another covalent modification occurs at two lysine residues close to the C-terminus, K217 and K219, which serve as substrate for the conjugation of small ubiquitin-related modifier 1 (SUMO1) in human cells (Xu et al., 2011). Mutating these residues to glutamic acid attenuates viruses in cell culture, but the mechanism is unclear (Xu et al., 2011). Both lysines are conserved in over 70% of avian NS1 sequences available on NCBI. In human and swine sequences, the most common amino acid at position 217 is glutamic acid (E217) in 46% of sequences, followed by lysine (K217) in 45%. K219 is conserved in 92% of human and swine isolates and lies within a C-terminal nuclear localization sequence found in the NS1 proteins of most strains (Greenspan et al., 1988; Melén et al., 2007).

One of the most striking examples of NS1 species-specific adaptation occurs at the very C-terminal end of the effector domain, in the structurally disordered tail between residues 208 and 230. The length of most avian and mammalian NS1 proteins is 230 amino acids, but the length of this tail varies. As mentioned above, this region contains a second NLS that is destroyed by natural truncations that sometimes occur in both avian and mammalian strains (Greenspan et al., 1988; Melén et al., 2007). The NS1 proteins of swine-origin pdm09(H1N1) strains, like A/California/07/2009 (H1N1), contain an 11-amino-acid truncation at its C-terminus, putting their total length at 219 amino acids. An isolate from a fatal human H5N1 infection, A/Vietnam/1203/2004 (H5N1), encodes an NS1 protein with a 10-amino-acid truncation at the C-terminus, which was absent from related co-circulating avian H5N1 strains and from earlier high-pathogenicity H5N1 human isolates like A/HongKong/156/97(H5N1). It appears that the secondary NLS is not essential for viral replication and the role of such truncations in host adaptation and pathogenicity remains unclear. In the late 1940s, circulating human seasonal H1N1 strains acquired a seven-amino-acid extension at the C-terminus, which was maintained in the progeny H2N2 and H3N2 lineages until it was lost in the 1980s (Hale et al., 2008b).

Amino acids 227–230 in avian NS1 isolates also contain a PDZ-binding motif (PBM). PDZ domains are structurally conserved protein domains that appear in many diverse proteins and recognize short peptide motifs to facilitate protein-protein interactions, similarly to the well-known Src-homology (SH2 and SH3) domains (Fanning and Anderson, 1996). PDZ domains are found in a variety of signaling proteins, tyrosine phosphatases, at neuronal synapses, and in association with the cytoskeleton where they participate in motor trafficking of protein complexes. They are divided into several families that bind to the carboxyl-terminus of various proteins bearing an appropriate sequence of amino acids (Nourry et al., 2003). One such consensus sequence bound by a family of closely-related PDZ domains is an (S/T)×V motif. Large-scale sequencing studies revealed the PBM in the last four amino acids of a majority of avian influenza A virus NS1 proteins (Obenauer et al., 2006; Thomas et al., 2011). The majority of circulating avian IAVs have NS1 proteins with a C-terminal consensus sequence ESxV. The majority of circulating human strains in the past decades share the C-terminal consensus sequence RSxV. The human RSxV motif has a lower affinity than ESxV for mammalian PDZ domain-containing proteins (Liu et al., 2010; Golebiewski et al., 2011). NS1 proteins with an “ESEV” PBM specifically associate with the PDZ proteins Scribble, Dlg1, MAGI-1, MAGI-2, and MAGI-3, while those with “RSKV” do not. Infection of A549 cells with a virus that expressed an NS1 protein with the ESEV PBM resulted in co-localization of NS1, Scribble, and Dlg1 within perinuclear puncta (Liu et al., 2010). Association with Scribble prevented apoptosis and increased viral replication compared to an “ESEA” point-mutant virus in HeLa cells. Infection of polarized MDCK cells with the “ESEV” virus additionally resulted in functional disruption of cellular tight junctions (Golebiewski et al., 2011). The consequences of this species-specific difference for viral fitness and pathogenicity are unclear, however. The introduction of “ESEV” sequence into the swine strain A/WSN/1933 (H1N1) increased virulence in mice in an interferon-independent manner (Jackson et al., 2008). Replacing “ESEV” with the human “RSKV” in A/Turkey/Italy/977/1999 (H7N1) had different effects in human, mouse, and duck cells *in vitro*. “RSKV” virus replicated more efficiently in human A549 cells and in duck embryonic fibroblasts, whereas in mouse 3T3 cells, “ESEV” virus replicated to higher titres (Soubies et al., 2010a). In live mice, the same “ESEV” virus was more virulent and lethal and induced orders of magnitude more type-I interferon in the lungs, but in duck intestines, the same recombinant strain with RSKV-NS1 replicated more efficiently and induced more transcription of interferon-stimulated genes than its “ESEV” counterpart (Soubies et al., 2010a; Volmer et al., 2011). Removing the “ESEV” motif from the NS1 of LPAI A/Turkey/Italy/977/1999 (H7N1) by truncation slightly increased the histopathology in infected chicken lungs without increased replication, and no pathology or replication differences were seen in ducks (Soubies et al., 2013). In the context of highly pathogenic H5N1, the addition of either the avian “ESEV” or the human “RSKV” motifs to the naturally truncated NS1 of A/Vietnam/1203/2004 (H5N1) did not affect its virulence or replication efficiency in mice and chickens (Zielecki et al., 2010).

More species-specific polymorphisms exist in NS1 than described here, but phylogenetic analyses to identify them have so far outpaced the experimental investigations of their significance. In the following sections, we discuss patterns of host-adaptation in two specific functions of NS1 – broad host protein synthesis suppression and interference in type I interferon signaling.

## NS1 Sequestration of Host CPSF4 – General Host Protein Synthesis Suppression

**Figure 2:**
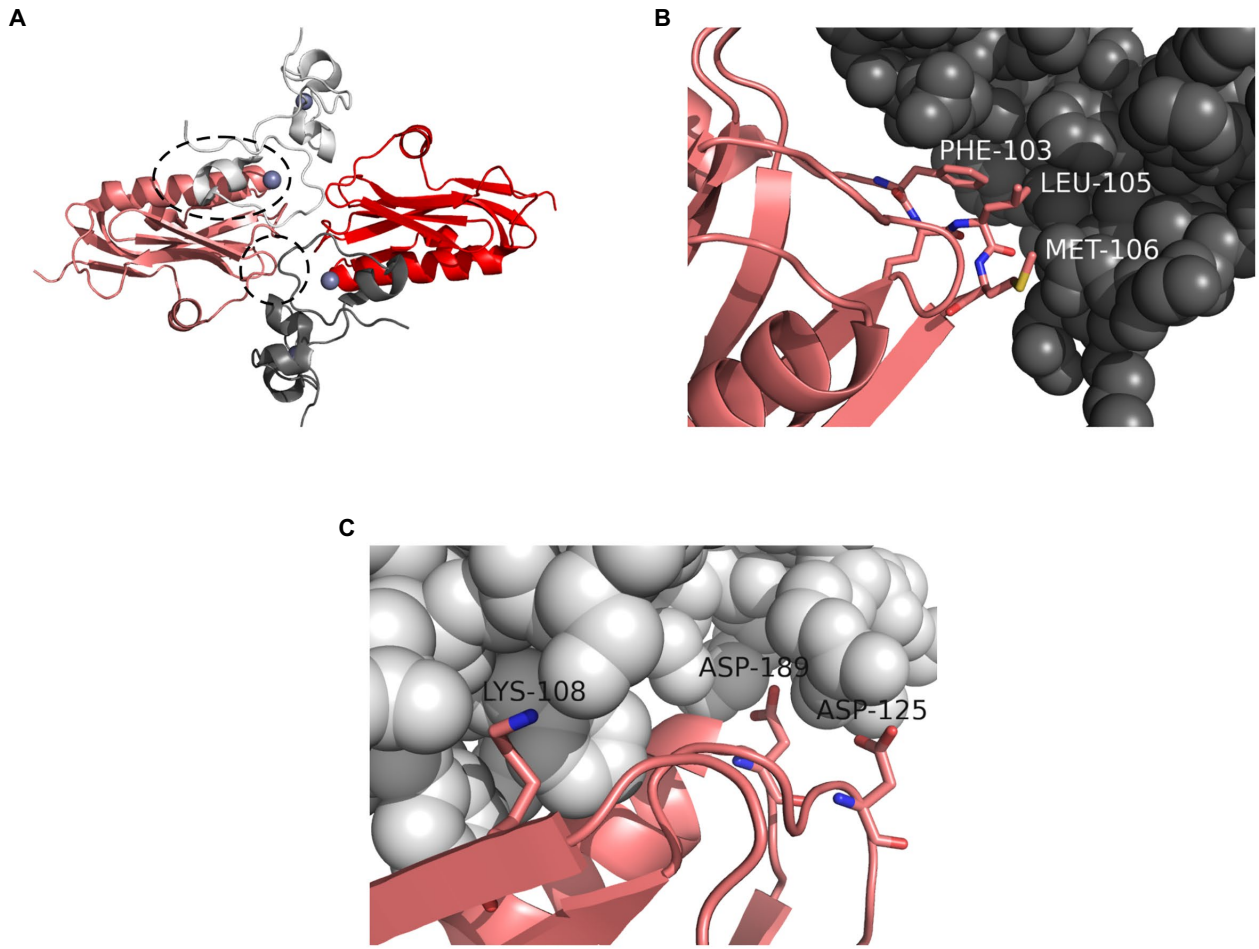
Interaction between NS1 and CPSF4. **(A)** Crystal structure of two NS1 effector domains (red and pink) in complex with two fragments of human CPSF4, each comprising the second and third zinc fingers (white and dark gray). Zinc atoms are shown as blue spheres. The major interaction interface between the pink NS1 chain and the white CPSF4 chain is highlighted with a dashed oval. The minor interaction interface between the pink NS1 chain and the dark gray CPSF4 chain is highlighted with a dashed circle. The structure is symmetrical. **(B)** A view of the minor interaction interface between NS1 (pink) and CPSF4 (dark gray) showing the contributions of NS1 residues F103 and M106. **(C)** A view of the major interaction interface between NS1 (pink) and CPSF4 (white) showing the contributions of NS1 residues K108, D125, and D189. All three residues are making polar contacts with CPSF4 (not shown). Image was prepared with PyMol using the crystal structure from Das et al. (2008; PDB ID: 2RHK).

Non-structural protein 1 broadly and non-specifically blocks host protein expression in infected cells by interacting with a component of host mRNA processing machinery, cleavage and polyadenylation specificity factor 4 (CPSF4, 30kDa subunit, also called CPSF30; Nemeroff et al., 1998; Noah et al., 2003; Das et al., 2008; Kuo and Krug, 2009; Hale et al., 2010). By sequestering CPSF4, NS1 prevents the proper 3'-end processing of host pre-mRNA species and their export from the nucleus. A crystal structure of NS1 in complex with a CPSF4 fragment is available (Das et al., 2008) and is congruent with NS1 mutagenesis studies (Hale et al., 2010; Steidle et al., 2010). NS1 proteins bind the second and third zinc fingers of CPSF4, which are perfectly conserved at the amino acid level between humans, pigs, chickens, ducks, and many other species. The interaction occurs through two separate interfaces, a minor interface involving NS1 residues F103 and M106 and a major interface involving NS1 residues K108, D125, and GGLEWND183–189 (Das et al., 2008; Hale et al., 2010; Figure 2A). Mutations at either of these interfaces abolish the NS1-CPSF4 interaction *in vivo* (Hale et al., 2010; Steidle et al., 2010). Examining the conservation of these amino acids among avian strains vs. human and swine strains available on the NCBI influenza database reveals an interesting inverse pattern. Residues 183–188 are universally conserved and help form a pocket at the major interface. In human and swine NS1 peptide sequences available on NCBI (46,737 full-length sequences, 9,508 nonredundant on May 23, 2021) residues F103 and M106 are conserved in over 96% of proteins (Figure 2B), but residues 108, 125, and 189 are less conserved (Figure 2C). K108 is conserved in only 51% of human and swine sequences, D189 also in 51%, and a D125E substitution occurs in 68% – a majority of human and swine isolates. The NS1 protein of A(H1N1)pdm09, which is of human origin, does not bind CPSF4 because of substitutions at these three poorly conserved residues (Hale et al., 2010). In avian NS1 sequences (22,285 full-length sequences, 7,181 nonredundant on May 23, 2021) the pattern is inverted. Residues K108, D125, and D189 are highly conserved, while residue F103 is conserved in only 66% of sequences, and residue M106 in 83%. The NS1 proteins of some avian viruses that infect humans, like A/Hong Kong/156/1997 (H5N1) and A/Shanghai/patient1/2013 (H7N9), do not interact with CPSF4 because of tandem substitutions at positions 103 and 106 (Dankar et al., 2013; Liu et al., 2013). It appears that CPSF4 binding is not strictly conserved in viruses of either host pool, but that different selective pressures act on different residues that make up the two interacting interfaces.

A broad suppression of host protein synthesis in infected cells could potentially benefit the virus in two ways – by liberating more cellular resources for viral protein synthesis and by repressing innate immune signaling and effector functions because mRNA is not being translated (Khaperskyy et al., 2014). The intuitive hypothesis is that losing the ability to bind CPSF4 would attenuate the virus (Noah et al., 2003). However, the case is not so straightforward because host factors are also required for efficient viral replication (Bouloy et al., 1978; Watanabe et al., 2010). In fact, some evidence suggests that loss of CPSF4 binding may be selected upon viral adaptation to new host species to enhance viral RNA replication and protein synthesis (Dankar et al., 2011; Forbes et al., 2012; Chauché et al., 2017). Two independent studies demonstrated that loss of function mutations at positions 103 and 106 are spontaneously selected when human A/Hong Kong/1/1968 (H3N2) is adapted to mice by serial passage (Dankar et al., 2011; Forbes et al., 2012). Dankar et al. (2011) showed that F103L and M105I mutations in NS1, in two different recombinant viral backgrounds, enhanced replication and virulence in BALB/c mice, enhanced viral protein synthesis in MDCK cells, and enhanced the ability to replicate in mouse cells pre-treated with interferon. Forbes et al. (2012) examined 12 different adapted variants that emerged from serial passage of human H3N2 in mice and found that, while eight of them lost the ability to inhibit CPSF4, they simultaneously increased interferon-beta antagonism and yielded equivalent or greater viral titres in the lungs compared to the wild-type starting virus. Mutations at M106 were independently selected in two of the serially passaged viruses. Such a mutation also appeared in a mallard H9N2 virus adapted to quail (Hossain et al., 2008). Another two of the emergent mouse-adapted variants in Forbes et al. (2012) selected mutations at position 125 (D125G), which also appears in a mouse-adapted variant of A/Aichi/2/1968 (H3N2; Narasaraju et al., 2009). Using a minigenome assay, Forbes et al. (2012) demonstrated an inverse relationship between functional CPSF4 binding and viral polymerase activity. They hypothesized that this effect is due to a lack of host transcription factors and host 5' mRNA caps to prime viral transcription when host pre-mRNA processing is suppressed.

The prototypical laboratory IAV strain PR8 has been adapted to mice over generations and is also deficient in CPSF4 binding due to tandem mutations at positions 103 and 106 in the minor interaction interface – F103S and M106I (Steidle et al., 2010). While loss of CPSF4 binding does not seem to impair interferon inhibition by NS1, it does seem to correlate with greater induction of pro-inflammatory cytokines, which in turn correlates with greater pathogenicity. Accordingly, wild-type PR8 and the experimentally adapted H3N2 variants described above are highly pathogenic in mice. Hale et al. (2010) showed that artificially restoring CPSF4 binding in the human A(H1N1)pdm09 NS1 resulted in faster clearance in mice and reduced titre in respiratory tracts of ferrets.

Equine influenza viruses offer another example of apparently adaptive loss of CPSF4 binding (Chauché et al., 2017). Examining equine H3N8 virus evolution since its spillover from birds in 1963, Chauché et al. (2017) found that the original avian NS1 protein was a competent CPSF4 binder, but progeny of that lineage in horses acquired an E186K mutation that abolished the interaction while enhancing the virus’ ability to control interferon through JAK/STAT signaling in equine cells.

It seems likely that, at least in certain cases, in the adaptation of an IAV to a new host environment where polymerase function may not yet be optimally adapted, it is advantageous to lose CPSF4 binding in order to enhance viral transcription and translation. However, this loss can also be accompanied by increased virulence, which can result in isolation of the incapacitated host and potentially decreased transmission. Thus, there may be an opposing selective pressure favouring the maintenance or re-emergence of this function in well-established endemic IAV pools. Clark et al. (2017) describe one example of the re-emergence of CPSF4 binding in human H1N1 strains. They show that currently circulating descendants of the A(H1N1)pdm09 strain have evolved to regain this ability with six amino acid substitutions that include E125D. Recombinant viruses bearing these NS1 proteins inhibited IFN and pro-inflammatory signaling more effectively than the original A(H1N1)pdm09 NS1 in mice and displayed a reduced disease severity. CPSF4 also appears to have a role in intron splicing and NS1 also inhibits this activity (Li et al., 2001). In particular, IAV infection affects the alternative splicing of the master regulatory transcription factor p53 (Dubois et al., 2019). It appears that the interaction between NS1 and CPSF4 may contribute to changing the balance of splicing, leaving the cell more amenable to viral replication. It should be noted that NS1 can also inhibit p53 transcriptional activity directly by preventing it from binding to its promoter site, potentially through direct protein-protein interaction (Terrier et al., 2013), but whether this blocks or enhances apoptosis is not yet clear (Wang et al., 2010; Yan et al., 2016).

CPSF4 binding, like most functions of NS1, is hard to examine in isolation because of the overlapping nature of NS1 interacting interfaces. For example, amino acid 125 and its neighbours also participate in an interaction with the antiviral effector PKR to suppress its activity (Min et al., 2007). In a mutagenesis study investigating PKR inhibition, the authors found that mutating this region of NS1 led to attenuation, but that in certain mutants the attenuation was offset by enhanced viral RNA replication at early timepoints (Min et al., 2007).

## NS1 Interference in the RIG-I Pathway – Inhibition of Interferon Signaling

A key function of NS1 proteins in human cells is the suppression of antiviral interferon signaling. By interfering with the RIG-I signaling pathway, a central pillar of early IAV detection, NS1 proteins block the induction of type I interferons (IFN-α and IFN-β) and the ensuing antiviral response (Gack et al., 2009; Koliopoulos et al., 2018). The virulence of 1918 pandemic influenza in experimentally infected macaques was associated with a marked lack of RIG-I signaling and transcription of interferon-stimulated genes (ISG; Kobasa et al., 2007). The pathogenesis of HPAI A/Hong Kong/156/1997 (H5N1) and its progeny was associated with the interferon resistance conferred by their NS1 proteins (Seo et al., 2002; Lipatov et al., 2005). There are at least three ways in which NS1 proteins potentially inhibit the signaling of the human RIG-I pathway: by sequestering dsRNA, by interacting directly with RIG-I, or by blocking TRIM25-mediated ubiquitination of RIG-I CARD domains. Early studies suggested that dsRNA binding was a key mechanism of innate immune suppression by NS1 (Lu et al., 1995; Ludwig et al., 2002). However, it appears that the affinity of NS1 for the dsRNA backbone is relatively weak and insufficient to competitively inhibit human RIG-I (Min and Krug, 2006; Krug, 2014). There is some evidence for high-affinity co-operative binding of NS1 RBDs to certain virus-specific RNA sequences (Marc et al., 2013), but there are also studies suggesting that very little free dsRNA actually accumulates within influenza A-infected cells (Pichlmair et al., 2006; Weber et al., 2006; Wisskirchen et al., 2011).

Non-structural protein 1 can interact with RIG-I CARD domains directly, in a strain-specific manner (Mibayashi et al., 2007; Dankar et al., 2013; Jureka et al., 2015). This interaction appears to involve residues in both the N-terminal RBD (Jureka et al., 2015) and residues 103 and 106 in the C-terminal effector domain (Dankar et al., 2013). Crude purifications from cells overexpressing recombinant proteins showed that NS1 could be found in insoluble fractions containing RIG-I and MAVS (Mibayashi et al., 2007). However, a more recent study by the same group, using bimolecular fluorescence complementarity and super-resolution microscopy showed that in fixed cells NS1 localizes to cytoplasmic foci that contain TRIM25 and RIG-I, but exclude MAVS, and that NS1 interacts to a greater extent with TRIM25 than with RIG-I in those cells (Sánchez-Aparicio et al., 2017).

**Figure 3:**
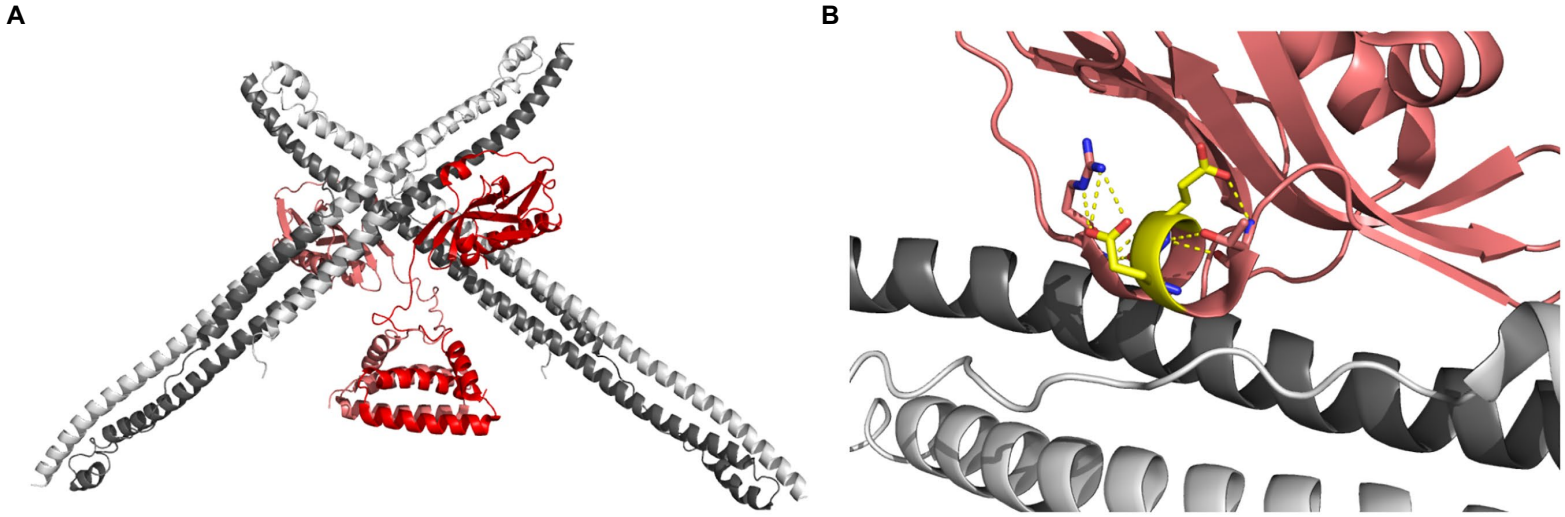
Interaction between NS1 and TRIM25. **(A)** Crystal structure of the NS1 proteins (red and pink) in complex with four fragments of human TRIM25, each comprising the coiled coil domain (white and dark gray). **(B)** A view of the NS1 residues E96 and E97 (colored yellow) facing away from TRIM25 and making polar contacts with neighbouring residues. Image was prepared with PyMol using the crystal structure from Koliopoulos et al. (2018; PDB ID: 5NT2).

Non-structural protein 1 binds human TRIM25 to prevent K63-linked ubiquitination of RIG-I CARD domains and thus destabilize the RIG-I/MAVS interaction (Gack et al., 2009; Koliopoulos et al., 2018; Woo et al., 2019). The interaction occurs with the long helical coiled-coil domain of TRIM25, which is also responsible for TRIM25 homo-dimerization. Antiparallel homo-dimerization is necessary for TRIM25 proteins to covalently attach K63-linked ubiquitin chains to target molecules (Sanchez et al., 2014, 2016). There are currently two different model mechanisms to explain how NS1 binding blocks TRIM25 enzymatic activity. The first model proposes that NS1 prevents TRIM25 dimerization (Gack et al., 2009). In the first report describing this interaction, using co-immunoprecipitation experiments, Gack et al. (2009) showed that NS1 binds to the coiled coil domains (CCD) of TRIM25 and prevents homo-multimerization. Using targeted mutagenesis, the authors also showed that NS1 residues R38, K41, E96, and E97 were essential for this interaction. The second model arises from a recent crystal structure of NS1 in complex with the TRIM25 CCD (Koliopoulos et al., 2018; Figure 3A). The authors of this crystal structure began by investigating the contributions of R38 and K41. It is known that full-length NS1 proteins bearing these wild-type residues aggregate in solution and precipitate out, necessitating tandem R38A/K41A mutations for crystallography (Bornholdt and Prasad, 2008; Carrillo et al., 2014; Koliopoulos et al., 2018). To address this, the authors used biolayer interferometry to determine that the C-terminal effector domain of NS1 is responsible for the interaction with TRIM25, and that full-length mutants bearing R38A and K41A mutations are still able to bind. Their crystal structure showed NS1 binding to already-formed CCD dimers and proposed that its position sterically inhibits ubiquitin ligation to a bound target by occupying the space where ubiquitin ligation would happen.

Notably, in this structure, NS1 residues E96 and E97 also do not participate in the interaction interface and make polar contacts only with adjacent residues within the NS1 protein (Figure 3B). E96/E97 are highly conserved across all influenza A strains and host species and their functional significance, though apparent through mutagenesis, is still unclear. They lie on the surface of the ED and do not appear involved with its core structural stability. They do not contribute to the interfaces of the two known NS1 ED dimer conformations (Bornholdt and Prasad, 2006; Hale et al., 2008a). A study investigating the mechanism of phosphoinositide 3-kinase activation by NS1 found that residues E96/E97 were essential for this function despite not contributing significantly to its interaction with the PI3K regulatory subunit. In that case, the authors hypothesized a tandem interaction with the catalytic subunit involving these residues. In the case of TRIM25, it may be that the available crystal structure also does not fully recapitulate the conformations or multiprotein complexes that occur *in vivo*. It is also notable that in the NS1-CCD crystal structure, while each NS1 effector domain is shown in complex with a pair of dimerized TRIM25 CCDs, most of the interaction interface involves only one CCD chain.

An investigation of several different NS1 proteins from mammalian and avian influenza A strains found host species-specific and strain-specific differences in their interactions with human, mouse, and chicken TRIM25 (Rajsbaum et al., 2012). In that study, all the tested NS1 proteins bound to human TRIM25, but only avian NS1 bound strongly to chicken TRIM25. Chickens are missing the RIG-I gene but possess the related MDA5 receptor and many other functional components of the RIG-I signaling pathway (Barber et al., 2010; Magor et al., 2013). In murine cells, NS1 proteins did not bind mouse TRIM25 and were unable to inhibit mouse RIG-I-CARD ubiquitination, but instead they inhibited another ubiquitin ligase involved in RIG-I activation, Riplet (Rajsbaum et al., 2012). In ducks, NS1 from strain A/Duck/Guangdong/212/2004 (H5N1) blocks Muscovy duck MDA5 signaling (Wei et al., 2014). It is not known whether this is achieved by blocking the ubiquitination of MDA5 CARD domains, or in another manner. Curiously, a recent study found that avian-origin NS1 proteins were more effective than their human counterparts at suppressing innate immunity in human cells (Monteagudo et al., 2019).

## The Activities of NS1 Impact the Adaptive Immune Response

Given the intimate crosstalk between innate and adaptive immune responses, it is not surprising that the activities of NS1 also impact adaptive immunity. The ability of NS1 to suppress innate immune signaling specifically, and its ability to shut down host cell transcription broadly, both appear to impact the magnitude and quality of the primary adaptive response and of immunological memory.

Innate immune cytokine signals fine-tune the adaptive response towards different pathogen types. In the context of viral infections, type I interferon signaling is essential for proper dendritic cell maturation (López et al., 2003) and for the functional priming of CD8^+^ and CD4^+^ T-cell responses (Curtsinger et al., 2005; Kolumam et al., 2005; Havenar-Daughton et al., 2006; Keppler and Aichele, 2011). *In vitro*, when human myeloid dendritic cells (DCs) were infected with IAV, the NS1 protein prevented induction of type I interferon and the transcription of genes associated with DC maturation and T-cell stimulation (Fernandez-Sesma et al., 2006). In multiple animal infection models, mutating or deleting NS1 enhanced the primary adaptive immune responses and increased the speed and efficacy of secondary memory responses to recombinant influenza A. Moltedo et al. (2009) found that, in mice infected with wild-type PR8 virus, replication could proceed in the lungs for up to 2days without apparent innate immune detection. Dendritic cell infiltration and T cell activation in the surrounding lymph nodes did not occur until a delayed inflammatory burst, but when mice were infected with a virus lacking NS1, all these processes began on the first day (Moltedo et al., 2009). Several studies have dissected *in vivo* adaptive responses to viruses bearing truncated or deleted NS1 proteins. In a study comparing mice immunized with either wild-type or NS1-truncated PR8 viruses, Mueller et al. (2010) found that the mutant viruses, though attenuated and deficient in replication, still induced long-lasting antibody responses and a more effective cellular memory. After initial immunization, the NS1-truncated viruses replicated much worse than wild-type PR8 in the recipient mice and activated fewer CD8^+^ T cells. However, these CD8^+^ T cells, when adoptively transferred to naïve mice, expanded to a greater extent, and conferred greater protection from subsequent viral challenge, compared to CD8^+^ T cells taken from wild-type PR8-immunized mice (Mueller et al., 2010). Ferko et al. (2004) also found an association between interferon induced by NS1 mutant viruses and the magnitude of the CD8^+^ memory response. The authors immunized mice with a panel of PR8 viruses bearing different NS1 truncations and subsequently challenged them with a high dose of wild-type PR8. They found that the quantity of CD8^+^ T cells in lymph nodes surrounding the challenged respiratory tracts, at 60h post challenge, were correlated with the levels of IFN-α/β induced during immunization (Ferko et al., 2004). A similar correlation was observed in pigtailed macaques. Baskin et al. (2007) immunized macaques intratracheally with either formalin-killed wild-type H1N1 virus (human influenza A/Texas/36/1991) or a live attenuated version of the same strain encoding only the first 126 amino acids of the NS1 protein. The live attenuated vaccine had a very low replication capacity with no observed microscopic lung pathology and minimal IAV antigen staining in tissues, but it induced a transient interferon burst; the killed vaccine induced less interferon. Upon homologous challenge with a live wild-type virus, the group that had been immunized with killed vaccine supported more viral replication and the secondary response was characterized by high interferon and inflammatory signaling and lower specific T cell and B cell responses. In contrast, the group that was vaccinated with the attenuated NS1-truncated virus, upon challenge, had a lower inflammatory burst and a more mature secondary response that drastically curbed viral replication (Baskin et al., 2007). NS1 truncation also improves heterosubtypic protection. When mice were immunized intranasally with either wild-type PR8 or with a mutant version encoding only the first 124 amino acids of NS1, the latter group were better protected against subsequent challenge with an H3N2 strain (Vasilyev et al., 2021). The mice that had been immunized with WT PR8 suffered 40% mortality when they were challenged with A/Aichi/2/68 (H3N2) whereas the mice immunized with the NS1-truncated virus had 100% survival and a secondary response characterized by lower proinflammatory signaling and improved CD8^+^ T cell responses (Vasilyev et al., 2021). Vaccinating pigs with an H3N2 live virus bearing a similar NS1 truncation conferred heterosubtypic protection against an H1N1 swine influenza strain (Richt et al., 2006). These data show that interferon induction upon first encounter with a virus makes a more effective memory response and that NS1, therefore, functions to weaken the adaptive response and immunological memory. A limitation of these studies is that large NS1 truncations make severely attenuated viruses and do not show which specific interactions of NS1 are responsible for adaptive immunity suppression. It is unclear too, whether suppression of the adaptive response is entirely a consequence of innate signaling disruption, or if there are specific interactions of NS1 with components of adaptive immunity.

A study employing point mutations in NS1 revealed a role for CPSF4 binding and general host transcriptional shutoff in modulating specific immunity (Dunagan et al., 2021). Dunagan et al. (2021) infected mice with different versions of the 2009 pandemic H1N1 virus A/California/04/2009. The wild-type strain, as described previously, encodes an NS1 protein that is incapable of shutting off host cell transcription by binding cellular CPSF4. To test the significance of this interaction for adaptive responses, the authors generated a recombinant version of this strain in which the CPSF4 interaction was rescued by three point mutations in the NS1 protein – R108K, E125D, and G189D. The mutant viruses with CPSF4 interaction restored had reduced lethality and induced slightly less IL-6 and IFN-γ secretion, as measured by ELISA of bronchoalveolar lavage fluid (type I interferon secretion was not measured). After infection of mice with these viruses, Dunagan et al. (2021) quantified virus-specific CD4^+^ and CD8^+^ T cells in mediastinal lymph nodes and in spleens using ELISpot assays. At 7days post-infection, viruses with CPSF4-competent NS1 proteins showed significantly lower IAV-specific T cell numbers in both lymph nodes and spleens, and also induced lower HA-specific IgG levels in serum at 21 dpi. To assess immunological memory, the authors quantified virus-specific T cells in spleens and HA-specific B cells in bone marrow at 84days post-infection and found lower numbers of both in mice infected with CPSF4-competent NS1 viruses. Mice from all groups in the study were fully protected from a homologous re-challenge with the same strain at 84 dpi, but when they received a heterologous challenge with a high dose of PR8, only the mice primed with wild-type A/California/04/2009 were fully protected, while among the mice primed with CPSF4-competent virus, 20–25% succumbed.

There is also accumulating evidence that the NS1 protein plays a role in suppressing antigen presentation by MHC class I to CD8^+^ T cells. As with dendritic cell activation, optimal antigen presentation during viral infections also depends on interferon signaling. The MHC class I genes contain an interferon-stimulated response element in their promoters that is conserved across species, and they are inducible by type I interferons (Zoller et al., 1992; Ting and Baldwin, 1993). Tisoncik et al. (2011) used a transcriptome microarray to compare human tracheobronchial epithelial cells infected with wild-type or NS1-truncated influenza viruses to show that full-length NS1 suppresses genes associated with MHC class I antigen presentation, including components of the immune proteasome and peptide-loading machinery. When human myeloid dendritic cells were infected with live AIV *in vitro*, their antigen presentation through MHC class I was several-fold weaker than that of mDCs exposed to heat-killed non-replicating AIV. Furthermore, live AIV infection also suppressed cross-presentation, on MHC class I, of unrelated viral antigen either from whole inactivated cytomegalovirus or from CMV pre-processed peptides. The authors observed no suppression of antigen expression to CD4^+^ T cells *via* MHC class II (Tisoncik et al., 2011), and it is interesting to note that, unlike class I, MHC class II is not upregulated by type I interferon signaling (Ling et al., 1985; Inaba et al., 1986). Thus, it seems that reduced MHC class I antigen presentation may also be a consequence of interferon antagonism by the NS1 protein, but this may not be the only mechanism at play. In a recent study, Menachery et al. (2018) found that in a human respiratory epithelium cell line, Calu-3, infection with influenza A/Vietnam/1203/2004 (H5N1) increased genomic DNA methylation along the sixth chromosome, particularly in the MHC locus. It was unclear whether this was targeted epigenetic modification induced directly by the virus, but the authors did not see a similar effect when they infected cells with influenza A/California/04/2009, the H1N1 pandemic strain whose NS1 protein does not interact with host CPSF4.

It is certain that dampening the adaptive immune response to conserved viral antigens would be paramount for adaptation in a new species or host population, and data are accumulating to show that the intracellular activities of NS1 contribute to this. What is not yet clear is whether this suppression of the primary adaptive and memory responses occurs solely because of innate signaling dysregulation and transcriptional shutoff, or if there are specifically targeted mechanisms by which NS1 antagonizes the adaptive arm of immunity.

## NS1 Evolution in a Broader Context

Influenza A virus NS1 evolution is complex because of the many overlapping and sometimes compensatory interactions in which the protein participates. It is even more complex when considered in context of the complete viral genome and the variety of species that can be infected. NS1 proteins do not evolve in isolation, but in the context of a particular IAV genome constellation. Certain mutations in NS1 co-vary with mutations in other viral proteins, like NA and PB2, that are associated with host and tissue specificity (Lopes et al., 2017). The segmented genome of IAVs and their ability to reassort greatly increases the genetic diversity and the adaptational possibilities. Taubenberger and Morens (2009) postulate that different viral genetic constellations can arrive at wholly different solutions to the problem of host switch and adaptation, depending on the specific context (Taubenberger and Morens, 2009). Conversely, the same virus may find different adaptations in hosts of different genotypes, even if they belong to the same species. This was illustrated in a study by Prokopyeva et al. (2016), where A(H1N1)pdm09 virus was adapted by serial passage in three different mouse lines in parallel and, while all achieved adaptation and lethality in their respective hosts, cross-challenge revealed that the variants were not always lethal in a different mouse strain. It is also hard to define a clearly adaptive phenotype, as illustrated by the ambiguous nature of the NS1:CPSF4 interaction. While experimental data suggest that a reduced ability to block host gene expression correlates with increased virulence, inflammation, and a more potent immunological memory response, observational studies show that, sometimes, a loss of CPSF4 interaction may still be selected. It is possible that efficient replication in a new host, the likelihood of the host’s survival, and the likelihood of viral transmission may impose opposing selective pressures that can be balanced differently in different situations. A case in point is the (albeit controversial) observation that highly pathogenic avian H5N1 strains arise primarily in domestic poultry and do not persist long-term in the wild aquatic avian reservoir (Kim et al., 2009; Krauss et al., 2016; Pantin-Jackwood et al., 2016). These highly pathogenic strains are typically not shed effectively from the cloacas of infected ducks (Keawcharoen et al., 2008; Vanderven et al., 2012). The problem of interpretation is further complicated by the artificial nature of laboratory adaptation studies, where deliberate serial passage virtually eliminates likelihood of transmission as a factor in viral evolution. The high infectious doses and routes of inoculation used in experimental infections may also not reflect how natural infections occur in the wild. Experimental infection studies do not mimic the conditions of viral evolution and are more well-suited to answering binary questions, but they can reveal functional principles that we can use to understand the dynamics of viral infection.

Selective pressures act in other dimensions besides protein function. For example, studies have shown that avian influenza virus genomes typically have a higher GC content than their swine and human counterparts, which reflects the host genome composition and declines upon adaptation of avian strains to mammals (Rabadan et al., 2006; Greenbaum et al., 2008; Dunham et al., 2009). The authors hypothesize that this shift in nucleotide composition is driven by selection for greater compatibility with host cofactors and the host cellular environment, but also suggest a possible contribution of RNA editing by the host as part of the antiviral response (Rabadan et al., 2006). Cao et al. (2018) investigated RNA editing in humans, quails, and chickens in response to infection with different strains of influenza A virus. Using transcriptome data from human tracheobronchial epithelial cells and from infected animal tissues, they discovered that RNA editing frequencies differed by subtype. They found that H1N1 and H3N2 infections in human cells upregulated the expression of adenosine deaminases acting on RNA (ADAR) enzymes and induced more adenine (A) to inosine (I) RNA editing events. In contrast, H5N1 infection in human cells did not induce significant changes in ADAR expression and A-to-I editing events. Expression of apolipoprotein B mRNA editing complexes (APOBECs) and the cytidine to uracil (C-to-U) editing events that they mediate remained comparable to controls across all three influenza subtype infections. Using transcriptome data from chickens and quail infected with H5N1 and H5N2 subtypes, the authors found no significant changes in RNA editing frequencies, which mirrored the results from H5N1-infected human cells. However, in this study the authors assessed RNA editing events in the host transcripts only and not in the viral transcripts, so it is still unclear to what extent these processes drive changes in viral genome composition during evolution in different hosts. Two regions in the nucleotide sequence of the NS genomic segment produce hairpins in their positive-sense transcripts with species-specific structural differences, the significance of which is still unclear (Vasin et al., 2016).

## Conclusion

It is clear from deletion and mutation studies that a functional NS1 protein is absolutely essential for successful IAV replication in any host. It follows that the ability of NS1 to productively interact with the immune system of a new host is mandatory for viral establishment and transmission. What is less clear is which of the many functions of NS1 are the essential ones under which conditions. The diversity of animal species that play host to IAVs, and the polymorphic nature of NS1 proteins both point to the fact that different solutions can exist to the problem of host immune evasion. In this review, we examined two prominent and well-studied functions of IAV NS1 proteins – the ability to non-specifically suppress host gene expression by interacting with CPSF4 and the ability to suppress interferon signaling by interacting with the RIG-I pattern recognition receptor pathway. Both mechanisms dampen innate immune signaling and both reduce the efficacy of the adaptive immune responses to the advantage of the virus. However, CPSF4 interaction appears to be expendable, or perhaps even disadvantageous in certain contexts, as evidenced by the abundance of non-interacting NS1 variants circulating in the wild among humans and animals. On the other hand, some form of interferon signaling inhibition appears to be essential in all cases.

In considering the role of NS1 in IAV evolution and species adaption, there are three big questions that should be addressed by a combination of observational studies and laboratory testing. The broadest question is how much NS1 adaptation is necessary for successful establishment in a new vertebrate host. Because of the complexity of the NS1 interactome and the importance of context, both host and viral, this question is hard to answer, but as our functional knowledge grows, we can formulate good testable hypotheses. For example, a highly pathogenic H5N1 strain could infect dogs but could not be transmitted to contact animals and did not cause serious disease (Giese et al., 2008). It would be interesting to ask whether the NS1 protein of this strain can interact with components of the canine RIG-I pathway. As global influenza surveillance efforts grow for both humans and wildlife, and as more sequence data become available, we are increasingly able to track viral evolution in real time and to spot patterns and commonalities that can then be examined in targeted functional studies. Innovative techniques like deep transcriptome sequencing and single-cell sequencing are being used to track viral mutation and host responses simultaneously (Yang et al., 2010; Ladell et al., 2013), and machine learning is being applied to predict viral evolution (Hie et al., 2021). Such approaches could elucidate how much selection acts on NS1 genes of IAV in different hosts. The second question is whether NS1 proteins participate in specific interactions that uniquely target adaptive immune responses. This question can be answered by mining NS1 interactome data for associations with components of adaptive immunity, to determine if it makes some specific protein-protein interactions or if adaptive response suppression occurs entirely as a consequence of interferon antagonism and transcriptional repression *via* CPSF4 and other transcription factors (Marazzi et al., 2012; Zhao et al., 2018). A third question, long overdue, is whether unique interactions, or a lack thereof, occur between NS1 and the components of the mallard duck immune system. There is a body of literature describing the disease resistance of mallard ducks as the natural hosts of IAV, and their rapid innate immune responses accompanied by persistent CD8^+^ T cell activation (Alexander et al., 1986; Cooley et al., 1989; Laudert et al., 1993; Kim et al., 2009; Cornelissen et al., 2013; Saito et al., 2018; Evseev and Magor, 2019). Some highly pathogenic H5N1 viruses are lethal to juvenile ducks at high doses (Pantin-Jackwood et al., 2012), and experimentally swapping NS1 genes appears to have little effect in such cases (Sarmento et al., 2010). However, adult mallard ducks are more disease resistant and tolerate higher infectious doses than other avian species (Pantin-Jackwood et al., 2012, 2013). In chickens, the lack of a RIG-I gene is predicted to make them profoundly susceptible to influenza disease (Barber et al., 2010). It would be valuable, therefore, to discover whether NS1 proteins interact with and suppress the RIG-I signaling pathway and interferon induction in mallard duck cells as they do in the cells of mice and men. This could be assessed by experiments testing protein-protein interactions between NS1 and duck RIG-I pathway components, like the RIG-I receptor itself and the ubiquitin ligases TRIM25 and Riplet (Rajsbaum et al., 2012). Functional characterization of the duck RIG-I pathway has begun in recent years (Miranzo-Navarro and Magor, 2014; Wei et al., 2014; Wu et al., 2014; Cheng et al., 2015; Xiao et al., 2018, 2020) and it will be insightful to learn whether the co-evolutionary history between this species and the ancestors of modern IAV has produced a different balance in the arms race between immunity and pathogen. Today, phylogenetic studies can identify host-specific signatures faster than they can be characterized and so our understanding of the nature of influenza host-adaptation is far from complete. Given the intriguing complexity of this process, we will require an integrated approach that includes cutting edge methods in cooperation with classical bench science.

## Author Contributions

DE: writing – original draft. DE and KM: writing – review and editing. All authors contributed to the article and approved the submitted version.

## Funding

We gratefully acknowledge QEII Graduate Scholarships (DE) and funding from CIHR project grant PJT 159442 (KM).

## Conflict of Interest

The authors declare that the research was conducted in the absence of any commercial or financial relationships that could be construed as a potential conflict of interest.

## Publisher’s Note

All claims expressed in this article are solely those of the authors and do not necessarily represent those of their affiliated organizations, or those of the publisher, the editors and the reviewers. Any product that may be evaluated in this article, or claim that may be made by its manufacturer, is not guaranteed or endorsed by the publisher.
